# Comment on “Facebook as a Novel Tool for Continuous Professional Education on Dementia: Pilot Randomized Controlled Trial”

**DOI:** 10.2196/21505

**Published:** 2020-10-30

**Authors:** Yusuke Saishoji, Akihiro Shiroshita, Yasushi Tsujimoto

**Affiliations:** 1 Department of General Internal Medicine National Hospital Organization Nagasaki Medical Center Omura Japan; 2 Department of Pulmonology Kameda Medical Center Kamogawa Japan; 3 Department of Nephrology and Dialysis Kyoritsu Hospital Kawanishi Japan; 4 Healthcare Epidemiology School of Public Health, Graduate School of Medicine Kyoto University Kyoto Japan

**Keywords:** internet-based intervention, education, spin bias

We read the article “Facebook as a Novel Tool for Continuous Professional Education on Dementia: Pilot Randomized Controlled Trial” by Chan et al [1] with great interest. The idea that face to face education is difficult and education via the internet or social networking systems is necessary is intriguing; this is an important perspective in the midst of the COVID-19 pandemic. In the article, the editor wrote, “readers are advised to carefully assess the validity of any potential explicit or implicit claims related to primary outcomes or effectiveness”; hence, we would like to discuss some perspectives.

First, the primary outcome was measured using the differences in the scores of the pre- and postintervention knowledge assessments, which comprised the 25-item Dementia Knowledge Assessment Scale (DKAS) and a formative evaluation of 20 multiple-choice questions. However, the authors’ conclusion is focused on the outcome of improving participants’ knowledge concerning a single subscale in DKAS. This interpretation might be a spin that could warp the interpretation of results and mislead readers [2].

Second, the article has issues with multiple testing. When tests are divided into subscales, some of them may have significant differences. To show a significant difference in the effect, corrections to the multiple tests are required [3].

Finally, this study is a pre and post study; therefore, a paired *t* test should be used instead of a two-sample *t* test. The two-sample *t* test estimates the treatment effect using only the responses at follow-up, and it does not use any information at baseline, which may be useful for increasing efficiency if the baseline and follow-up outcomes are correlated [4].

## Abbreviations

DKAS: Dementia Knowledge Assessment Scale

## References

[1] Chan Windy Sy, Leung Angela Ym (2020). Facebook as a Novel Tool for Continuous Professional Education on Dementia: Pilot Randomized Controlled Trial. J Med Internet Res.

[2] Boutron Isabelle, Dutton Susan, Ravaud Philippe, Altman Douglas G (2010). Reporting and interpretation of randomized controlled trials with statistically nonsignificant results for primary outcomes. JAMA.

[3] Li Guowei, Taljaard Monica, Van den Heuvel Edwin R, Levine Mitchell Ah, Cook Deborah J, Wells George A, Devereaux Philip J, Thabane Lehana (2017). An introduction to multiplicity issues in clinical trials: the what, why, when and how. Int J Epidemiol.

[4] Yang L, Tsiatis AA (2001). Efficiency Study of Estimators for a Treatment Effect in a Pretest–Posttest Trial. The American Statistician.

